# Supplementary material for: Are carbon emissions trading and green financial instruments synergistic? -Comprehensive quantitative research based on content analysis
Source: PLoS One. 2024 Mar 7;19(3):e0298601. doi: 10.1371/journal.pone.0298601 (PMC10919601; doi:10.1371/journal.pone.0298601)
Supplement: S1 Data — (ZIP) [file pone.0298601.s001.zip › Data/地级市Z_F工作报告.zip!/╣π╢1⁄2/╤⌠╜¡╩╨/└┤╫╘╣1⁄2╓┌║┼í╛┬φ┐╦╩2╛▌═°í┐_2015.docx]

各位代表：

　　我代表市人民政府向大会作政府工作报告，请予审议，并请政协各位委员和其他列席人员提出意见。

　　2014年工作回顾

　　2014年是我市实现经济发展三大目标之后，在新的起点上加快发展取得新成效的一年。市政府在省委、省政府和市委的正确领导下，在市人大及其常委会和市政协的监督支持下，全面贯彻落实中央和省的决策部署，紧紧围绕主题主线和“在加快发展中转变方式，实现幸福追赶”的核心任务，坚持稳中求进工作总基调，主动适应经济发展新常态，深入实施“双化”驱动、蓝色崛起和融入珠三角战略，全力稳增长、调结构、促改革、惠民生、防风险，经济社会持续健康较快发展，总体完成了市六届人大四次会议确定的目标任务。

　　一、着力稳增长，经济保持平稳较快发展

　　积极应对经济下行压力，努力促进经济在合理区间运行，全市实现地区生产总值1168.55亿元，增长10.5%，增速居全省第三;人均生产总值46938元，增长9.9%;地方公共财政预算收入62.95亿元，增长17.2%;规模以上工业总产值1865.78亿元、增加值467.57亿元，分别增长18.1%和18.7%，增加值增速居全省第一;工业用电量65.69亿千瓦时，增长18.6%。发挥投资拉动效应，固定资产投资662.01亿元，增长16.7%，其中77个重点项目完成投资246.9亿元，增长6.5%。拓宽民间投资领域和范围，民间投资424.84亿元，增长18.8%。努力扩大消费需求，社会消费品零售总额581.9亿元，增长10.4%。出台措施支持外贸稳定增长，进出口总额26.9亿美元，增长12.9%。发挥金融在稳增长中的作用，通过发行企业债等形式融资38.36亿元投入基础设施建设。引导金融机构加大对实体经济的支持，金融机构人民币贷款余额694.79亿元，增长32.4%。扶持民营经济发展，规模以上民营工业增加值增长21.5%，民营经济对经济增长贡献率达83.3%。

　　二、坚持调结构，产业转型升级步伐加快

　　加快经济结构调整，三次产业比例调整为17.5∶50.4∶32.1。推动产业对接融入珠三角，我市加入珠中江经济圈，纳入珠江西岸先进装备制造产业带。加快工业结构调整，培育发展电力能源、特种钢、先进装备制造等新兴产业，广青金属科技有限公司等3家企业纳入国家行业规范，英格电气等项目建成投产，阳江核电站2—6号机组等项目扎实推进。改造提升传统产业，五金刀剪产业总产值510.03亿元，增长16%。培育发展大型骨干企业，新增主营业务收入超10亿元企业5家、超50亿元企业1家。开展“1+10”专业招商，新引进国叶云计算智慧城等项目102个，总投资372.65亿元，其中超10亿元项目12个。加强与珠海合作共建，促进产业园区扩能增效，4个省级园区规模以上工业总产值803亿元，增长29%，占全市规模以上工业总产值43%。积极发展现代农业，阳东区省级现代农业示范区建设顺利，全市新增省级以上农业龙头企业4家、市级现代农业园区15个，农业总产值329.94亿元，增长4%。沙扒国家一级渔港通过农业部验收，渔业总产值149.04亿元，增长4.7%，水产品产量118.45万吨，增长3.4%。加快发展现代服务业，我市加入“高铁+游轮”中国岸线旅游联盟，海陵岛以总分第一名获“广东十大美丽海岛”称号，大角湾海上丝路旅游区通过国家5A级景区景观质量评审，“南海Ⅰ号”全面保护发掘进展顺利，全市接待游客1498.2万人次，旅游总收入153.6亿元，分别增长19.3%和34.5%。阳江港开通集装箱内贸运输航班，全市首个电商产业园投入运营。提高科技创新能力，制定发布五金刀剪国家标准1项，新增国家级高新技术企业2家、省级名牌产品11个。

　　三、注重抓统筹，新型城镇化扎实推进

　　以中心城区扩容提质为带动，统筹推进以人为核心的新型城镇化。全面推进滨海新区建设，核心区发展规划优化提升，一批商住小区陆续建成，江城一小城南校区、城南污水处理厂(一期)竣工，东门南路等一批市政道路建成通车。编制中心城区扩容提质建设规划，市区创业路北段等市政工程完成，盈信广场等旧城改造项目进展顺利。阳东区正式挂牌成立。成功创建国家园林城市，创建国家卫生城市和国家环保模范城市全面推进。加快县城扩容和旧城改造，推进中心镇改革发展，城镇面貌明显改观，产业和人口进一步聚集。启动美丽乡村建设，阳东区省级新农村示范片建设扎实开展，全市2个镇、6条村被省认定为第一批名镇名村，4条村被评为省第三批宜居示范村。 加强城乡基础设施建设，深茂铁路阳江段、汕湛高速公路阳春段动工建设，云阳高速公路阳春段加快推进，海陵岛大桥等项目前期工作扎实有效，省道277线海陵大堤改建等项目建成，新农村公路硬底化任务超额完成。220千伏登高输变电工程投入运营，234个农村电网改造项目完成。加固达标海堤50公里，除险加固水库22宗，改造灌区23宗。建成无线局域网络热点(WLAN)1109个，城镇无线宽带网络覆盖率达100%。加强生态环境保护，造林7.67万亩，淘汰燃煤锅炉44台、黄标车5098辆，单位生产总值能耗下降3.66%。

　　四、积极促改革，发展活力明显增强

　　加大简政放权力度，市、县两级新一轮政府机构改革基本完成，撤销市级议事机构165个，新取消和下放行政审批事项26项。推进经济领域重点改革，工商登记实现“先照后证”，新登记市场主体1.8万户，其中有限责任公司2405户，增长55.7%。企业投资管理体制改革加快，社会信用和市场监管体系建设不断加强。市属国有企业治理结构进一步完善。创新事业单位管理体制，下放市直部分事业单位管理权限，推动事业单位建立法人治理结构，组建了市应急指挥中心，推进计生服务机构与妇幼保健机构整合，在县级公立医院开展综合改革试点。市区居民生活用水阶梯水价改革完成。加快社会组织改革，组建了市社会组织联合会，新增社会组织283家。深化农村改革，开展农村土地承包经营权确权登记颁证试点，41条行政村政经分离改革完成。

　　五、努力惠民生，社会事业全面进步

　　开展基本公共服务均等化综合改革，加快发展以民生为重点的社会事业。十件民生实事顺利完成，全市公共财政民生支出71.5亿元，增长7.9%。稳定和扩大就业，新增城镇就业4.25万人，转移农村劳动力5万人，城镇登记失业率2.4%。完善社会保障体系，城镇职工五大险种参保人数138.44万人次，增长5.39%，企业退休人员月平均养老金、城乡居民养老和医疗保险待遇、低保五保、孤儿供养保障、残疾人生活津贴等标准全面提高。市养老院、市残疾人辅助器具资源中心建成使用。建成公租房565套、渔民上岸安居房400套，改造农村低收入住房困难户住房7701户、棚户区住房110户，建成因灾“全倒户”新居164户。加快教育事业发展，全市新增省教育强镇(街道)24个，江城区创建为省教育强区，达到国家义务教育发展均衡县(市、区)评估认定标准。市高级技工学校挂牌成立阳江技师学院。实施文体服务提升工程，市国民体质监测中心、阳江漆艺基地建成使用，基层文化站(室)提前一年实现全覆盖，举办了群众文化节、风筝创新大赛等文体活动400多场。加强医疗卫生工作，江城区人民医院由市人民医院托管整合，市人民医院新住院大楼加快建设，人感染H7N9禽流感等重大疾病有效防控。有序实施“单独两孩”政策，低生育水平保持稳定。扎实推进新一轮扶贫开发“双到”工作，有劳动能力贫困户人均纯收入提高40%。开展“社会矛盾化解年”活动，信访形势保持平稳。开展打击涉黄赌毒、电信诈骗犯罪等六大专项行动，社会治安持续稳定，人民群众安全感和满意度不断提高。食品药品监管加强，安全生产形势保持稳定。双拥优抚政策全面落实，军政军民更加团结。工会、妇女儿童、关工委、慈善、老龄事业持续发展，民族宗教、外事侨务、统计、供销、档案、打私、人防、气象、地震等工作取得新进步。

　　六、深入转作风，政府自身建设有效加强

　　扎实开展市政府党组和政府系统党的群众路线教育实践活动，严格执行中央八项规定，认真查摆整改“四风”突出问题，干部队伍密切联系群众加强，调查研究、文风会风明显改进，厉行节约反对浪费形成良好风气。深入开展“服务效率提升年”活动，推动机关作风明察暗访常态化，“庸懒散奢”等不良风气有效整治。加强网上办事大厅建设，基本实现市、县、镇、村四级网上办事大厅全覆盖。自觉接受人大、政协监督，定期向市人大、市政协报告和通报工作，办理人大代表建议65件、政协委员建议案和提案共163件。密切与各民主党派、无党派人士和各人民团体的联系，进一步深化政务公开、网络问政，充分听取社会各界的意见和建议。推进依法行政，制定实施专家咨询论证等重大行政决策制度，依法行政考评在粤东西北地区排第三名。严格落实党风廉政建设责任制，强化审计监督，严肃查处一批违法违纪案件。

　　各位代表，过去一年的成绩来之不易，这是市委正确领导、科学决策的结果，是市人大和市政协有效监督、大力支持的结果，是全市人民团结奋进、努力拼搏的结果。我代表市人民政府，向全市人民及所有关心支持阳江建设发展的同志们、朋友们，表示衷心的感谢!

　　我们也清醒地看到，我市经济社会发展还存在一些困难和问题。主要是：经济下行压力较大，主要经济指标增速放缓;产业结构不优，新兴产业和现代服务业发展滞后;重大项目和大型骨干企业不多，部分企业生产经营困难;城镇化水平不高，中心城区辐射带动能力不强;环境容量指标趋紧，节能减排力度有待加大;基本公共服务水平不高，保障和改善民生任务艰巨;一些单位和公职人员服务意识不强、工作效能不高，作风建设仍需继续深化。对此，我们将高度重视，采取措施认真解决。

　　2015年工作安排

　　2015年是全面深化改革的关键之年，是全面推进依法治国的开局之年，也是全面完成“十二五”规划的收官之年。市政府工作的总体思路是：全面贯彻落实党的十八大、十八届三中、四中全会、中央经济工作会议、习近平总书记系列重要讲话精神和省委十一届四次全会精神，按照市委六届八次全会的部署要求，紧紧围绕主题主线和“在加快发展中转变方式，实现幸福追赶”的核心任务，主动适应经济发展新常态，坚持稳中求进工作总基调，以提高经济发展质量和效益为中心，深入实施“双化”驱动、蓝色崛起和融入珠三角战略，突出“三大抓手”，加快转型升级，狠抓改革攻坚，统筹城乡发展，加强民生保障，推进依法行政，完成“十二五”发展目标，规划“十三五”发展任务，努力推动经济平稳健康较快发展和社会和谐稳定。

　　全年主要预期目标是：生产总值增长10%左右，并力争更高的水平;规模以上工业总产值增长17%;固定资产投资增长13%;进出口总额增长3%;社会消费品零售总额增长11%;地方公共财政预算收入增长12%;居民消费价格涨幅控制在3%左右;居民收入增长与经济增长基本同步;城镇登记失业率控制在3%以内;节能减排约束性指标完成省下达年度计划。重点抓好八个方面工作：

　　一、促进经济平稳健康较快发展

　　抓好重点项目建设。全年安排重点建设项目86个，总投资2132亿元，年度投资238亿元;预备重点项目58个，总投资1346亿元。完善项目协调推进机制，解决征地拆迁、资金筹措等方面的困难和问题，保障项目顺利实施。推动阳江核电站2号机组等项目投产，促进阳江抽水蓄能电站等项目加快进度，推进国叶云计算智慧城等项目开工建设，加快阳春新兴铸管等项目前期工作。做好“十三五”重点项目谋划和前期工作，争取更多项目纳入国家、省的计划安排。

　　加快交通等重要基础设施建设。全年完成交通项目投资30亿元。加快深茂铁路阳江段、云阳高速公路阳春段、汕湛高速公路阳春段等15个续建项目建设，推进海陵岛大桥、国道325线北惯至白沙段改线等12个项目动工，做好阳东至阳江港沿海高速公路(含阳江港大桥)等9个项目前期工作。加快500千伏回隆、220千伏旗鼓岭输变电工程建设，促进漠阳江双捷引水工程拦河闸重建、漠阳江中下游综合治理等水利工程尽快开工。

　　提高招商引资实效。切实抓好“1+10”专业招商和先进装备制造业招商，发挥资源和产业优势，着力引进有资金实力、有品牌、有团队的大企业项目。加大招商推介力度，加强与珠海等地区联合招商，扎实开展乡贤反哺招商、以商引商、中介招商，拓展境外招商。算好项目投入、产值、税收、用地和排放“五笔账”，严把项目效益关和环保关。

　　积极培育消费新增长点。落实国家和省扩大内需政策，创造消费良好条件。加强与大型电商平台合作，加快阳江电子商务创业园建设，推动县域电子商务发展，建设农村电商体系。编制智慧城市建设总体规划，加快宽带网络、移动通信、三网融合等信息基础设施建设。拓展信息消费领域，增强信息产品供给能力，促进数字家庭应用消费。鼓励和支持社会力量进入养老服务业领域，加快发展养老服务业。组织企业北上西行，拓展阳江产品外销市场。加强农超对接，促进农村消费。深化收入分配制度改革，提高城乡居民收入和消费能力。做好市场保障和价格稳定工作，整顿和规范市场价格秩序。

　　二、加快产业转型升级

　　推进产业园区扩能增效。加快珠海(阳江)产业转移园等园区基础设施建设，完善科技研发、仓储物流等服务配套。开展园区信息化建设，促进信息化与工业化融合发展。加大项目建设力度，推动省级园区做大做强主导产业，增强产业集聚能力，力争新增主营业务收入超50亿元企业1家、超10亿元企业3家。推动市县共建园区融入省级园区产业集聚区，成为区域产业布局重要组成部分。清理闲置土地和厂房，促进土地节约集约利用。

　　推动工业扩量提质。优化沿海临港工业发展规划，强化临港工业项目用地用海保障，推进阳江海洋经济特色产业基地和高新区临港工业园建设。围绕省建设珠江西岸先进装备制造产业带，引进国内外大型龙头骨干企业和“高精专”中小型配套企业，突出发展关键基础件和原材料制造，建设华南地区重要基础原材料生产基地。立足港口优势和产业基础，发展游艇、风电、船舶与海洋工程等先进装备制造业。加快广青200万吨金属压延项目建设，延伸不锈钢产业链，打造中国(阳江)高端不锈钢产业基地。大力发展新能源、新材料、生物医药等新兴产业。开展新一轮技术改造，加大技改投资和政策扶持力度，支持企业智能化改造、设备更新和公共服务平台建设，推动五金刀剪、食品加工等传统产业优化升级。扶持中小微企业健康发展，促进民营经济发展上水平。

　　大力发展现代服务业。围绕创建国家5A级旅游景区，抓好海陵岛旅游综合开发，增强高端旅游景区的综合带动能力。推进阳春生态旅游发展，加快特色旅游强镇建设，创建一批3A、4A级景区(点)。加强区域旅游合作，不断拓展旅游市场。强化旅游景区管理，大力整治环境卫生及占道经营、非法营运等影响景区形象行为。优化城乡商业网点，统筹布局开发城市综合体，构建完善的商贸服务体系。规划建设汽车城、农海产品批发等专业市场。大力发展现代物流业，加快保税仓储物流基地等项目建设，引进和培育现代物流龙头企业，构筑区域性现代物流中心。积极发展金融业，引进国内外金融机构在阳江设立分支机构。依托阳江漆艺、风筝等非物质文化遗产发展特色文化产业。培育发展研发设计、体育健身等新兴服务业。

　　扎实推进农业现代化。深化农村体制改革，全面铺开农村土地承包经营权确权登记颁证，引导和规范土地有序流转，加快培育新型农业经营主体，发展多种形式的适度规模经营。加强国家级万亩粮食高产创建示范片建设，推进阳东区省级现代农业示范区建设，创建10个市级现代农业园区。加快渔船更新改造，发展100艘以上钢质渔船。推进南鹏列岛、青洲岛等深水网箱产业园区建设，促进深水网箱养殖技术升级。发展水海产品深加工业，提高产品附加值。加快渔港升级，争取闸坡中心渔港纳入高标准现代化渔港建设，申报溪头、对岸渔港为国家一级渔港。

　　增强科技创新能力。推进科技体制改革，完善技术创新市场导向机制，促进金融科技产业融合发展。建立健全多主体协同创新机制，强化企业技术创新主体地位，支持大型骨干企业与高校、科研院所开展产学研合作，加强专业镇中小微企业科技服务平台建设。积极培育发展新型研发机构，建设阳江五金刀剪产业技术研究院，推动增材制造(3D打印)重大科技专项实施。深入开展质量强市工作，加快国家刀剪质检中心与德国莱茵公司检测合作项目建设，组建中国阳江(五金刀剪)知识产权快速维权中心。加强科技人才培养，积极引进科技领军人才和团队。

　　三、加强开放合作

　　稳定外贸增长。落实国家和省稳定出口的政策措施，调动企业扩大出口积极性。培育优势企业和出口名牌，提高五金刀剪等传统产品出口竞争力，加大水海产品出口扶持力度，扩大高新技术产品出口。鼓励企业参加境外展会，巩固欧美等传统市场，拓展俄罗斯等新兴市场。加强先进装备、技术和资源性材料进口，扩大进口规模。

　　加强与珠海合作共建。进一步增强主体意识，积极做好服务工作，促进对口帮扶工作全覆盖。以产业园区共建为重点，开展“项目建设年”活动，推进风电装备、纸业、金属压延、港口物流等招商，力促明阳风电、维达纸业等项目开工，推动粤裕丰钢铁、中国纸业阳江造纸基地落户。加快阳江港码头建设和合山机场改扩建。开通珠海阳江游艇旅游，打造“珠阳港澳”海上黄金旅游线路。加强人才培训交流，推进百家学校、医院结对共建，建成粤德合作阳江职业教育培训基地。加强两地区县(市)合作共建，推动两地区县(市)成立合资公司，联合推进园区、交通、旅游等方面建设，提升县域经济发展水平。

　　深化区域合作。积极参与珠三角地区分工合作，加快融入珠三角。加强与珠中江经济圈城市规划、交通建设衔接，深化金融、改革、科技、社会建设等领域合作。推进湛茂阳临港经济带共建，加强与港澳台地区交流，积极参与21世纪海上丝绸之路建设，着力构建全方位、多层次、宽领域的区域合作新格局。

　　四、抓好新型城镇化建设

　　加快滨海新区起步区建设。扎实推进金平路、城南东路、城南西路等主干道路建设。加快完善新区功能配套，建成市妇幼保健院新院、幼儿园等一批公共服务设施，抓好漠阳湖公园等城市景观带建设。坚持产城融合发展，加大招商和项目建设力度，规划建设高铁商务聚集区、国际金融大厦等亮点工程，加快产业和人口聚集。积极探索市场化投入机制，多渠道解决滨海新区建设融资问题。

　　推进中心城区扩容提质。完成市新型城镇化等规划编制。统筹市区功能布局，推动阳东城区东进南拓，加快道路、水电、通信等基础设施建设，促进基本公共服务均等化发展，提升阳东城镇化水平，让阳东发展得更好。启动全市“三旧”改造规划修编，加大旧城改造力度。加快市区南山公园、气象公园等市政工程建设，认真落实“三个一”环境卫生整治制度，推进国家卫生城市和国家环保模范城市创建。加强和改进城市管理，优先发展公共交通，推进市政管网建设改造，抓好老旧街区环境整治，妥善解决各类“城市病”。

　　抓好中心镇改革发展。深化简政强镇事权改革，加大审批事项下放力度，扩大充实基层政府事权和财权。开展中心镇新一轮总体规划修编。探索市场化融资模式，拓展中心镇建设资金渠道。引导中心镇因地制宜发展特色产业，抓好城镇基础设施建设，加快城乡公共服务发展，不断提升城镇化水平。

　　推进美丽乡村建设。落实美丽乡村建设专项资金。深入实施美丽乡村建设“四大工程”，加快完善农村道路、水电和信息化等基础设施，深入整治农村人居环境，确保120条美丽乡村全部通过验收。推进阳东区省级新农村示范片建设，继续开展“宜居城镇”、“宜居村庄”示范评选和名镇名村申报。

　　五、深化重点领域改革

　　推进行政审批和事业单位改革。进一步加大简政放权力度，按照省的部署再取消和调整一批审批事项，全面清理非行政许可审批事项。优化再造行政审批流程及行政审批标准化建设，加快行政审批事中、事后监管制度建设。编制政府部门权责清单，一季度公布第一批试点单位的权责清单。完善网上办事大厅，推进企业专属网页和市民个人网页建设。深化事业单位法人治理结构改革，在部分事业单位实行全员聘任制改革。完成全市党政机关公务用车制度改革。

　　深化财政金融体制改革。根据省深化财税体制改革总体方案部署，加快建立政府事权和财政支出责任相适应的运行机制，深化预算、民生财政保障、财政投融资等制度改革。优化财政收入结构，不断提高税收收入比重。加强政府债务管理，加快建立规范的政府举债融资机制。落实我市在“新三板”、“新四板”挂牌的企业优惠政策，推动企业上市融资。大力发展农村普惠金融，完成阳西县农村普惠金融试点工作，发展支农特色小额贷款公司。优化金融生态，防范金融风险。继续深化国有企业改革，积极发展混合所有制经济。

　　加快工商登记和投资管理体制改革。完善宽进严管配套措施，探索实行工商营业执照、组织机构代码证和税务登记证“三证合一”登记制度，逐步实现工商登记注册标准化。加快实施企业投资项目准入负面清单、审批标准化清单和监管清单。深化公共资源交易体制改革，推动公共资源向各类投资主体公平配置。加快社会信用和市场监管体系建设，构建统一规范的市场主体信息公示体系。

　　加强社会管理创新。培育发展社会组织，加大财税扶持力度，引导社会组织完善内部治理结构，提升社会组织承接政府职能转移能力，扩大政府向社会组织购买服务。推进新型智库建设。深化户籍制度改革，有序推进农业转移人口市民化。创新基层社会治理，推进社区网格化服务管理。加快培育社工人才，发展壮大志愿者队伍。

　　六、持续保障和改善民生

　　办好十件民生实事。围绕人民群众最关心的热点难点问题，精心组织实施十件民生实事，不断提高人民群众生活水平和幸福指数。

　　强化就业和社会保障。实施更加积极的就业政策，促进高校毕业生、异地务工人员、就业困难人员等重点群体就业。加强职业技能培训，促进劳动力转移就业。继续推进社保扩面征缴和五保低保扩面提标，稳步提高养老、医疗保险和五保低保等待遇水平，全面实施基本医疗保险市级统筹。加强保障性住房建设，新建公租房766套，改造农村低收入住房困难户住房5000户。推进市、县残疾人综合服务设施和养老服务“五个一”工程建设，加快阳春、阳西、阳东养老院规划建设。

　　大力发展社会事业。抓好创建教育强市工作，完成43个教育强镇(街道)创建任务，确保阳春、阳东和阳西创建为教育强县(市、区)，实现创建省教育强市目标，推进国家义务教育发展基本均衡县(市)创建。实施特殊教育提升计划，推动各县(市、区)建成符合国家标准的特殊教育学校并投入使用。大力发展学前教育，扶持和规范民办教育，推进高等教育、职业教育、继续教育、社区教育健康发展。提高基本公共卫生服务质量，推进市区和镇级公立医院资源整合，深化县级公立医院综合改革，提高基本药物使用比例，减轻群众就医负担。加快市人民医院新住院大楼建设，推进卫生信息化平台建设，落实基层医疗卫生机构“五个一”医疗设备配置。抓好重大传染病防控。稳定低生育水平。加快发展文化体育事业，推进公共图书馆、文化馆免费向社会开放，整合资源建设市、县、镇三级文化体育综合服务示范中心(点)。加强地方特色文艺创演，开展形式多样的文化体育惠民活动。以“南海Ⅰ号”史迹参与申报海上丝绸之路世界文化遗产。全面完成新一轮扶贫开发“双到”目标任务。抓好双拥共建工作，巩固军政军民团结。认真解决妇儿发展重点难点问题。继续做好民族宗教、外事侨务、人防、档案、气象、打私等工作。

　　促进社会更加和谐稳定。做好“6+3”社会矛盾化解工作，切实解决群众合法合理诉求。推进阳光信访建设，实行网上受理信访制度。继续深化专项打击整治行动，依法打击和防范各类违法犯罪活动。落实一村(社区)一法律顾问制度，实施法治惠民工程。加强重点行业领域安全生产专项整治，坚决预防和遏制重特大安全事故。抓好农产品质量安全监管，强化食品药品专项整治。完善突发事件应急处置机制，提高预警和应急处置能力。依法加强网络安全管理。做好网络问政工作。

　　七、加强生态环境保护

　　抓好节能减排。严格控制高能耗、高污染企业落户，加快淘汰落后产能。发展循环经济，支持企业开展清洁生产技术改造，推动产业转移园申报为省循环经济园区。加快既有建筑节能改造，大力发展绿色建筑。抓好工业效能提升和节能改造，推广节能新技术、新产品使用。

　　加强环境整治。抓好漠阳江流域整治，保护好母亲河。加强农业面源污染防治，重点做好畜禽养殖污染整治。加快春湾镇等11个中心镇生活污水处理厂建设，推进一般镇生态型污水处理设施建设。严格执行环境空气质量新标准，划定禁止使用高污染燃料区域，加强脱硫脱硝设施监管，加快淘汰“黄标车”、燃煤锅炉，抓好重点月份和特殊天气空气质量监控，严格控制扬尘，有效控制PM10、PM2.5浓度。

　　构筑生态屏障。科学合理划定森林、林地、湿地、物种等林业生态红线。加强公园规划管理，严格实施公园绿线规划。推进新一轮绿化阳江大行动，抓好森林碳汇、生态景观林带、森林进城围城、乡村绿化美化等生态工程建设，重点完成阳江森林公园建设和打造阳阳高速公路生态景观林带示范段。加强海洋资源环境保护，抓好红树林自然保护区和湿地公园保护建设。

　　八、推进法治政府建设

　　加强民主法治建设。维护宪法尊严和权威，把遵守宪法和法律作为施政根本原则。认真落实人大及其常委会各项决议决定，定期向人大及其常委会报告工作，自觉接受监督。积极支持人民政协履行政治协商、民主监督、参政议政职能。认真办好人大代表议案、建议和政协委员提案。广泛听取各民主党派、工商联、无党派人士和各人民团体意见。推行政府法律顾问制度，落实公众参与、专家论证、风险评估、合法性审查、集体讨论决定等重大行政决策法定程序，探索建立重大行政决策终身责任追究和责任倒查制度。

　　提高依法行政水平。依法全面履行政府职责，坚持法定职责必须为，法无授权不可为。深化行政执法体制改革，坚持严格规范公正文明执法，全面落实行政执法责任制，加强执法监督，坚决排除对执法活动的非法干预。按照中央和省的部署，稳妥推进司法体制改革。坚持政府常务会议会前学法制度，加强公务员教育管理，运用法治思维和法治方式推动经济社会发展。完成“六五”普法任务，实行谁执法谁普法责任制，逐步建立以案释法制度。

　　加强行政权力运行制约。强化对权力集中部门和岗位的内部流程控制，防止权力滥用。完善政府内部层级监督和专门监督，建立常态化监督制度。加大审计工作力度，对公共资金、国有资产、国有资源和领导干部履行经济责任情况实行审计全覆盖。全面推进政务公开，重点抓好财政预算、公共资源配置等领域信息公开，加强农村集体“三资”管理服务平台建设，让权力在阳光下运行。

　　扎实转变政府作风。严格执行中央八项规定，大力整治“四风”问题和“庸懒散奢”行为，巩固提升党的群众路线教育实践活动成果，推进作风建设常态化。弘扬求真务实工作作风，做好服务企业工作，清理规范涉企行政事业性收费，积极帮助企业解决实际困难和问题，为企业发展创造良好环境。开展“工作落实年”活动，不断提高工作效能。健全惩治和预防腐败体系，完善廉政风险防控机制，严肃查处违法违纪案件，坚决纠正损害人民群众利益不正之风。

　　各位代表!阳江发展前景美好，幸福追赶需快马加鞭。让我们紧密团结在以习近平同志为总书记的党中央周围，在省委、省政府和市委的正确领导下，以奋发有为的精神状态，以改革创新的勇气锐气，凝心聚力，真抓实干，为阳江在粤东西北地区率先全面建成小康社会而努力奋斗!
